# COVID-19 does not influence functional status after ARDS therapy

**DOI:** 10.1186/s13054-023-04330-y

**Published:** 2023-02-05

**Authors:** Alice Bernard, Lina Maria Serna-Higuita, Peter Martus, Valbona Mirakaj, Michael Koeppen, Alexander Zarbock, Gernot Marx, Christian Putensen, Peter Rosenberger, Helene Anna Haeberle

**Affiliations:** 1 Department of Anesthesiology and Intensive Care Medicine, Tübingen University Hospital, Universitätsklinikum Tübingen, Hoppe-Seyler-Straße 3, 72076 Tübingen, Germany; 2 Institute for Clinical Epidemiology and Applied Biometry, Faculty of Medicine, University of Tübingen, Tübingen, Germany; 3 Department of Anesthesiology, Intensive Care and Pain Medicine, University of Münster, Münster, Germany; 4 Department of Intensive Care Medicine, University Hospital RWTH Aachen, Aachen, Germany; 5 Department of Anesthesiology and Intensive Care Medicine, University Hospital Bonn, Bonn, Germany

**Keywords:** COVID-19, Barthel index, Health-related quality of life, Viral pneumonia

## Abstract

**Rationale:**

Health-related quality of life after surviving acute respiratory distress syndrome has come into focus in recent years, especially during the coronavirus disease 2019 pandemic.

**Objectives:**

A total of 144 patients with acute respiratory distress syndrome caused by COVID-19 or of other origin were recruited in a randomized multicenter trial.

**Methods:**

Clinical data during intensive care treatment and data up to 180 days after study inclusion were collected. Changes in the Sequential Organ Failure Assessment score were used to quantify disease severity. Disability was assessed using the Barthel index on days 1, 28, 90, and 180.

**Measurements:**

Mortality rate and morbidity after 180 days were compared between patients with and without COVID-19. Independent risk factors associated with high disability were identified using a binary logistic regression.

**Main results:**

The SOFA score at day 5 was an independent risk factor for high disability in both groups, and score dynamic within the first 5 days significantly impacted disability in the non-COVID group. Mortality after 180 days and impairment measured by the Barthel index did not differ between patients with and without COVID-19.

**Conclusions:**

Resolution of organ dysfunction within the first 5 days significantly impacts long-term morbidity. Acute respiratory distress syndrome caused by COVID-19 was not associated with increased mortality or morbidity.

**Supplementary Information:**

The online version contains supplementary material available at 10.1186/s13054-023-04330-y.

## Introduction

Acute respiratory distress syndrome (ARDS) causes a significant reduction in long-term health quality; however, in most ARDS studies, the primary endpoint is patient mortality in the beginning.

With improvements in intensive-care treatment, health-related quality of life (HRQoL) after survival of ARDS came into focus in the recent decade, and more than 200 instruments have been defined to assess HRQoL [1–4]. Several studies, except for two, described no significant impact of ARDS on HRQoL compared to other ICU patients 6 months or 1 year after intensive care [5–10] before the COVID-19 pandemic. There is, however, conflicting data regarding this [11, 12]. Recently, several studies have determined HRQoL using different tools and defined the significant factors influencing HRQoL. The Barthel index (BI), for instance, is an established tool for assessing functionality in everyday-life and has previously been used in investigating outcome after ARDS therapy. It encloses everyday tasks such as dressing, body hygiene, and mobility, among other aspects [13].

To make long-term improvements for ARDS patients, rather than looking at the endpoint of a very differentiated disease pattern, the impact of single patient characteristics during ARDS therapy may help to interfere in advance, avoiding low HRQoL afterward. Therefore, we firstly strived to identify factors during intensive-care treatment of ARDS which significantly and independently influence functionality after  ICU  discharge, and can be used as outcome predictors in the future. Secondly, said factor or factors might not only serve as predictors, but could provide the chance to alter treatment and improve functionality after survival of ARDS.

The recovery of patients and the quality of life after intensive care therapy for ARDS have even gained significant public interest during the coronavirus disease 2019 (COVID-19) pandemic due to the large number of patients requiring ARDS therapy.

In this context, the question arises if ARDS based on COVID-19 is associated with a higher mortality or morbidity than ARDS of other origins, or if it’s “*just another ARDS*”.

## Materials and methods

### Study design and patients

The present study was a secondary analysis of the ThIlo trial, a prospective randomized multicenter trial assessing the efficacy of inhaled iloprost for the prevention of the development and progression of ARDS in critically ill patients. Study design and recruitment are described elsewhere [14]. The study was approved by the Institutional Review Board of the Research Ethics Committee of the University of Tübingen (899/2018AMG1) and the corresponding ethical review boards of all the participating centers. The trial was approved by the Federal Institute for Drugs and Medical Devices (BfArM, EudraCT No. 2016-003168-37) and registered at clinicaltrials.gov (NCT03111212). All patients provided written informed consent prior to enrollment.

Iloprost treatment did not show any significant effect on the outcomes of these patients; therefore, a secondary analysis was performed.

### Data collection

In addition to baseline information, the SOFA score was collected at baseline and on days 1, 2, 3, 4, 5, and 15 after enrollment. The BI was used to evaluate the functional status. The BI was determined by telephone calls or patient interviews at baseline and on days 28, 90, and 180 after study inclusion. The patients who died were assigned a score of 0. The BI scale was categorized into five groups (1) total dependency: 0–20 points, (2) severe dependency: BI 21–60 points, (3) moderate dependency: 61–90 points, (4) slight dependency: BI 91–99, and (5) independence 100 points [17, 18].

### Outcomes measures

The primary endpoint was disability at day 180, which was evaluated using the BI. The secondary endpoints included changes in the BI on days 28, 90, and 180 stratified by the etiology of ARDS (COVID-19 vs. non-COVID) and overall mortality at day 180. High disability was defined by a BI of 0–60 (total and severe dependency), and low/moderate disability by a BI of 61–100 (moderate/slight dependence and independence). Secondary endpoints included changes in the BI on days 28, 90, and 180 stratified by the etiology of ARDS (COVID-19 vs. non-COVID-19), change in SOFA score within 15 days follow-up and day 180 overall mortality.

### Power calculation

The ThIlo trial was powered for the primary endpoint (effectiveness of iloprost in ARDS). In this secondary analysis, a post-hoc power calculation was done to estimate the risk of high disability at day 180. The power calculation was based on a binary logistic regression model. An observed group size of 144 patients (144 with COVID-19 and 44 without COVID-19) was used for the power calculation. The outcome of high disability, which occurred in 57% of patients, requires an OR of 3.1 (or OR: 0.32) to achieve a power of 80% with an alpha of 5%. The calculation was performed using the software PASS 2020.

### Statistical analysis

All reported *P*-values were two-sided, and the significance level was set at ≤ 0.05. All statistical analysis was done using R statistical software version 4.1 and the program for statistical social sciences IBM SPSS software version 27.0 (IBM, New York, NY, USA).

Categorical variables are expressed as absolute and relative frequencies. Quantitative variables are expressed as means and standard deviations or medians and interquartile ranges according to the distribution of the data. Normality of the distribution was assessed by investigating skewness and kurtosis as well as QQ graphs and histograms. Categorical variables were compared using X^2^ tests (or Fisher´s exact test for small datasets).

Continuous variables were compared using an independent sample Student’s t-test for normally distributed data or the Mann–Whitney *U* test for non-normally distributed data.

Changes in the BI at baseline and after were evaluated using the nonparametric analysis for longitudinal Data “nparLD” (R-software) [19, 20].

### Identification of independent risk factors

Binary logistic regression was performed to evaluate independent risk factors for high disability by BI 180 days after study inclusion.

Candidate risk factors for the multivariate model were selected based on clinical reasoning and the statistically significant results of the bivariate analyses. Multicollinearity was checked using matrix correlation and variable inflation factors (VIF). Backward selection was used to remove the variables from the model sequentially. In addition, model comparisons were made using the log likelihood test (nested models), calibration was assessed with the Hosmer–Lemeshow goodness-of-fit test, and the area under the receiver operating characteristic curve (AUC) was used to examine the discrimination ability. The best fit model was used as final model. The results of the univariate and multivariate analyses are presented as odds ratios (OR), 95% confidence intervals (CI), and *P*-values.

### Analysis of SOFA score dynamic

Changes in the BI at baseline, 28, 90 and 180 days after enrollment and ARDS aetiologies groups (COVID-19 vs. others) were evaluated using the nonparametric analysis for longitudinal Data “nparLD” (R-software) [19, 20].

Temporal changes in the SOFA score values during the first 14 days after enrollment were analyzed by linear mixed model with random intercept. For this, BI high disability vs. low/moderate disability at day 180, ARDS etiologies (COVID-19 vs. others) and the interaction term “BI: Covid”, were entered as fixed effect variables.

### Survival analysis

Overall survival was analyzed using the Kaplan–Meier method, and the log-rank test was used to test differences in survival curves.

### Missing data

Multiple imputations were used to replace missing data with plausible values based on observed data [15] using fully conditional specification (multivariate imputation by chained equations). The following variables were included as predictors: age, sex, body mass index, SOFA score on day 5, presence of COVID-19, acute kidney injury, extracorporeal membrane oxygenation (ECMO), mortality, and BI on day 180. We used Rubin's combining rules to combine parameter estimates from models fitted using each complete dataset [16]. Using multiple imputation, the following information were generated: BMI (for 2 patients), hypertension (for 7 patients), SOFA at day 0 (for 15 patients), SOFA at day 5 (for 22 patients), positive blood cultures (for 1 patient), viral infection (for 1 patient), bacterial infection (for 1 patient), fungal infection (for 1 patient), time in the ICU (for 1 patient). In total, we created 50 complete datasets.

## Results

### Patient demographics

**Fig. 1:**
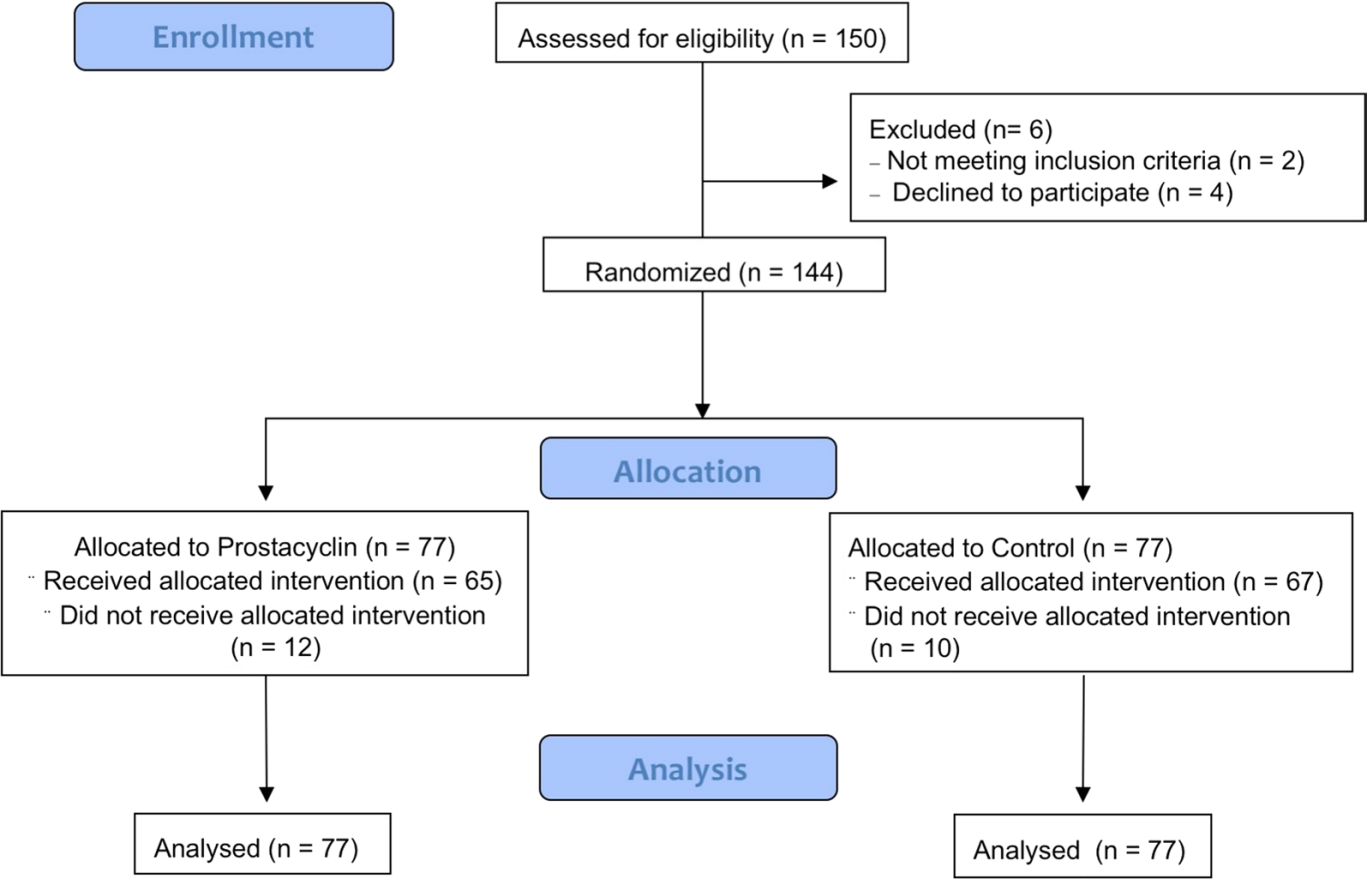
Flowchart of the recruitment process and the follow-up process of the ThIlo trial

Between July 2019 and May 2021, 150 patients were recruited for the ThIlo trial. Six patients were excluded, so that all in all, data of 144 patients were analyzed. The mean age was 58.5 ± 14.4 years, and 75% of the patients were male. The median length of ICU stay was 14 days (range 9–28 days). One hundred (69.4%) cases were classified as COVID-19 ARDS and 44 (30.5%) as non-COVID-19 ARDS. Treatment with iloprost did not show any significant effect on the outcomes. Figure 1 visualizes the recruitment process and the follow-up of our cohort.

Table 1 presents the baseline characteristics of the recruited patients according to the origin of ARDS (COVID-19 vs. non-COVID-19). Demographic characteristics were well-balanced between the groups. Patients with COVID-19 were treated with steroids (*n* = 74), IL-6-specific antibodies (*n* = 23), and remdesivir (*n* = 50). Additionally, there was no significant difference in disease severity at onset, as expressed by the SOFA score at baseline and day 5 and the minimum and maximum Horovitz indices (paO_2_/FiO_2_). Notably, the frequency of ECMO therapy was significantly higher in patients with COVID-19, as well as the incidence of additional viral infections and pulmonary bacterial superinfection pneumonia. The incidence of bacterial or fungal infections was similar in both groups.

**Table 1:** Baseline characteristics of patients

	n	COVID-19 ARDS (*n* = 100)	Non-COVID-19 ARDS (*n* = 44)	*P*-value
*Variables at baseline assessment*				
Gender	144			0.30^Chi2^
Female		22 (22.0%)	14 (31.8%)	
Male		78 (78.0%)	30 (68.2%)	
Age in years mean ($\pm$SD)	144	59.4 ($\pm$14.3)	56.6 ($\pm$14.6)	0.28^TT^
BMI median (IQR)	142	29.2 (26.3–33.9)	28.4 (24.4–33.6)	0.26^ MW^
Diabetes yes *n* (%)	141	33 (34%)	8 (18.2%)	0.086^Chi2^
Pulmonary diseases yes *n* (%)	144	22 (22%)	9 (20.4%)	0.99^Chi2^
Hypertension yes *n* (%)	137	54 (55.1%)	16 (41.0%)	0.19^Chi2^
Chronic kidney disease yes *n* (%)	144	4 (4%)	5 (11.4%)	0.19^F^
Min PaO_2_/FiO_2_ mean ($\pm$SD)	144	152.5 ($\pm$74.6)	146.6 ($\pm$59.1)	0.65^TT^
Max PaO_2_/FiO_2_ mean ($\pm$SD)	144	234.1 ($\pm$96.4)	264.6 ($\pm$95.6)	0.08^TT^
SOFA baseline mean ($\pm$SD)	129	10.8 ($\pm$2.9)	11.1 ($\pm$4.2)	0.68^TT^
SOFA after 5 days mean ($\pm$SD)	122	8.8 ($\pm$4.0)	9.3 ($\pm 5$.0)	0.53^TT^
Max FiO_2_/day				
Max P max mean ($\pm$SD)	140	28.4 ($\pm$4.5)	27.3 ($\pm$8.0)	0.29^TT^
Max P mean mean ($\pm$SD)	132	20.5 ($\pm$4.0)	20.0 ($\pm$6.4)	0.61^TT^
Max compliance mean ($\pm$SD)	109	58.1 ($\pm$25.1)	56.9 ($\pm$18.9)	0.83^TT^
Driving pressure mean ($\pm$SD)	124	12.4 ($\pm$4.2)	13.3 ($\pm$4.4)	0.29^ T^
*Variables during the follow-up*				
ECMO yes *n* (%)	144	32 (32%)	6 (13.6%)	0.036^Chi2^
Duration of ECMO median (IQR)	38	18 (9.5–41)	13 (9.5–20.7)	0.42^ MW^
Acute Kidney Insufficiency *n* (%)	144	24 (24%)	15 (34.1%)	0.293^Chi2^
CRRT yes *n* (%)	144	20 (20%)	15 (34.1%)	0.109^Chi2^
Positive blood culture* *n* (%)	143	31 (31%)	10 (23.3%)	0.46^Chi2^
Pneumonia *n* (%)	143	91 (91%)	30 (69.8%)	0.003^Chi2^
Bacterial infection *n* (%)	143	78 (78%)	30 (69.8%)	0.40^Chi2^
Viral infection *n* (%)	143	83 (83%)	18 (41.9%)	< 0.001^Chi2^
Fungal infection *n* (%)	143	48 (48%)	15 (34.9%)	0.21^Chi2^
Therapy	144			0.86^Chi2^
NaCl *n* (%)		51 (51%)	21 (47.7%)	
Ilosprost *n* (%)		49 (49%)	23 (52.3%)	
Time ICU days	143	13 (8–21)	16 (11–34)	0.040^ MW^

*TT* student T test independent samples, *F* fisher test, *MW* Mann Whitney U Test, *Chi2* Chi square test, *CRRT* continuous renal replacement therapy, *ECMO* extracorporeal membrane oxygenation, *AKI* acute kidney injury

^*^Any infections-positive blood culture

### Primary endpoint

#### Risk factors related to high disability

Univariate analysis (binary logistic regression) showed that age (OR = 1.04; 95% CI 1.01–1.07; *P* = 0.021), SOFA score at day 5 (OR = 1.17; 95% CI 1.06–1.3; *P* = 0.003), ECMO therapy (OR 2.51, 95% CI 1.02–6.18; *P* = 0.047), acute kidney insufficiency (OR = 2.50, CI 1.07–5.82, *P* = 0.036), and continuous renal replacement therapy (OR 2.79, CI 1.14–6.81, *P* = 0.026) were significant predictors for high or moderate disability according to the BI on day 180. Female sex was a protective factor against high disability (OR = 0.42, CI 0.19–1.07, *P* = 0.021). However, iloprost therapy (*P* = 0.89), history of pulmonary disease (*P* = 0.94), SOFA score at baseline (*P* = 0.064), infection (*P* = 0.48), and length of ICU stay (*P* = 0.24) did not have a significant impact on disability on day 180 (Table 2).

**Table 2:** Risk factors for high disability* after 180 days (univariate analysis using binary logistic regression after multiple imputation) (*n* = 144)

	OR	95% CI	P
*Variables at baseline assessment*			
Gender			
Male	1		
Female	0.42	0.19–0.91	0.031
Age in years	1.04	1.01–1.07	0.021
BMI	0.97	0.91–1.04	0.43
Diabetes yes	0.71	0.29–1.72	0.45
Pulmonary diseases yes	1.04	0.38–2.81	0.94
HTA yes	0.92	0.44–1.94	0.84
Min PaO_2_/FiO_2_	0.99	0.99–1.001	0.132
Max PaO_2_/FiO_2_	0.996	0.99–1.000	0.059
SOFA baseline	1.12	0.99–1.26	0.064
COVID-19	0.94	0.38–2.33	0.89
*Variables during the follow-up*			
SOFA 5 days ICU	1.17	1.06–1.30	0.003
ECMO yes	2.51	1.02–6.18	0.047
Acute Kidney Insufficiency yes	2.50	1.07–5.82	0.036
CRRT yes	2.79	1.14–6.81	0.026
Any infection yes	0.63	0.18–2.23	0.48
Viral infection yes	0.84	0.33–2.11	0.71
Therapy			
NaCl	1		
Ilosprost	1.06	0.48–2.32	0.89
Time ICU days	1.01	0.99–1.03	0.24

*CRRT* continuous renal replacement therapy, *ECMO* extracorporeal membrane oxygenation, *AKI* acute kidney injury

*High disability: Barthel index 0–60

Multivariable Cox regression showed that age, SOFA score on day 5 after enrollment, and ECMO therapy were the only independent variables associated with a moderate/high disability status 180 days after enrollment (Additional file 2: Table S1). The SOFA score on day 5 increased the odds of presenting with moderate/high disability at day 180 by 12% (OR = 1.12, 95% CI 1.01–1.98, *P* = 0.043). The area under the ROC curve (AUC) for 180-day disability probability was moderate for the SOFA score on day 5 (0.672, 95% CI 0.561–0.784) (Additional file 1: Fig. S1).

### Secondary endpoints

#### COVID-19 is not associated with a higher 180-day mortality

**Fig. 2:**
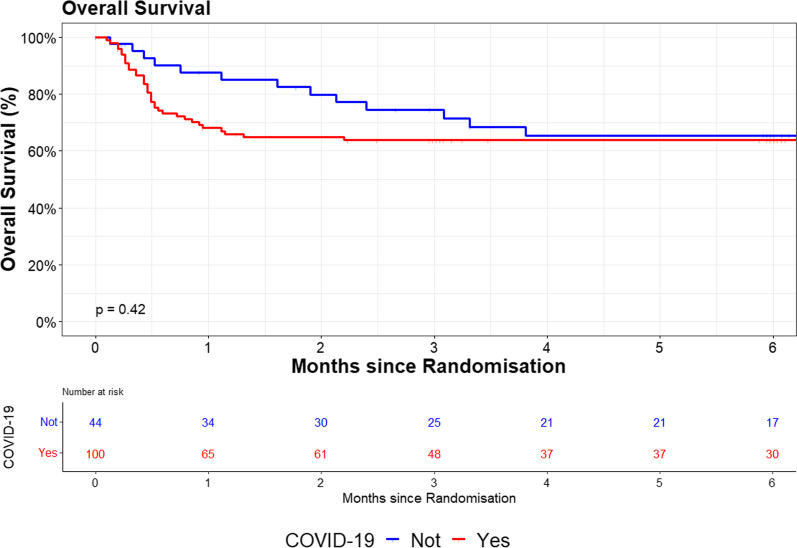
Survival rates of patients with COVID-19 ARDS (*n* = 100) and non-COVID ARDS (*n* = 44) until 180 days after recruitment do not differ between groups

During follow-up, 48 patients died; the overall survival at 30, 60, and 90 days was 73.9%, 69.3%, and 67.1%, respectively. Figure 2 shows the Kaplan–Meier survival analysis between the non-COVID-19 and COVID-19 patients. There was no difference in the 180-day mortality between the groups (log-rank test, *P* = 0.42).

#### COVID-19 is not associated with a risk of higher disability after ARDS

**Fig. 3:**
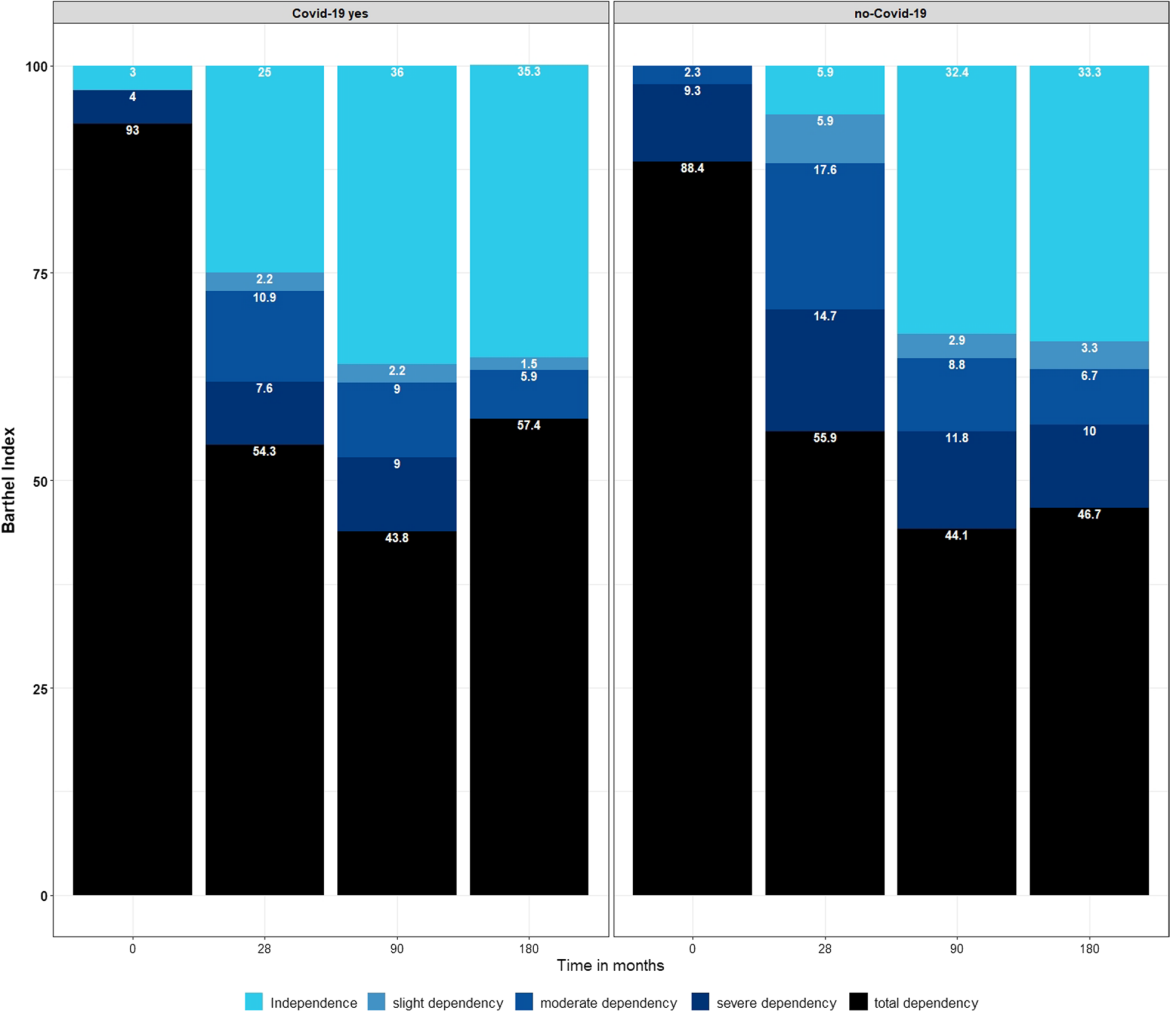
Proportions of Barthel indices at indicated time points (0, 28, 90, and 180 days) of patients with COVID-19 ARDS versus non-COVID ARDS. Total dependency: BI 0–20; severe dependency: BI 21–60; moderate dependency: BI 61–90; slight dependency: BI 91–99 and independence BI: 100. [baseline: *n* = 100 (COVID-19) and *n* = 43 patients (non-COVID); day 28: *n* = 92 (COVID-19) and *n* = 34 (non-COVID); day 90: *n* = 89 (COVID-19) and *n* = 34 (non-COVID); day 180: *n* = 68 (COVID-19) and *n* = 30 (non-COVID)] ( proposed by Shah et al. [21])

Table 3 shows the BI categorized by total, severe, moderate, slight dependence and independence. At baseline, 91.6% of the patients were found to have total dependency based on the BI since they were sedated at inclusion. In the overall study population, on days 28, 90, and 180, high disability was observed in 54.8%, 43.9%, and 54.1%, respectively. The percentage of high disability at day 180 was 57.4% in the COVID-19 group and 46.7% in the non-COVID-19 group. Figure 3 shows the proportion of the BI categorized by disability and stratified by COVID-19 ARDS and non-COVID ARDS over the time course (baseline, 28, 90, and 180 days). The analysis of variance- type test reveals no significant difference between the BI between the COVID-19 and non-COVID-19 groups at any time point (F = 0.08, *P* = 0.82). Time as an isolated parameter did have a significant effect on the BI (F = 0.64.9, *P* < 0.001); COVID-19 in relation to time did not show any significance (Interaction COVID*time: F = 0.82, *P* = 0.51) (Additional file 3: Table S2).

**Table 3:** Barthel Index categories at baseline and after follow-up (*n* = 143)

	*n*	Total cohort (*n* = 143)	*n*	COVID-19 ARDS (*n* = 100)	*n*	Non-COVID-19 ARDS (*n* = 43)
Barthel index baseline	143		100		43	
Independence (100) *n* (%)		3 (2.1%)		3 (3.0%)		0 (0%)
Slight dependency (91–99) *n* %)		0 (0%)		0 (0%)		0 (0%)
Moderate dependency (61–90) *n* (%)		1 (0.7%)		0 (0%)		1 (2.3%)
Severe dependency (21–60) *n* (%)		8 (5.6%)		4 (4.0%)		4 (9.3%)
Total dependency (0–20) *n* (%)		131 (91.6%)		93 (93.0%)		38 (88.4%)
Barthel index 28 days	126		92		34	
Independence (100) *n* (%)		25 (19.8%)		23 (25%)		2 (5.9%)
Slight dependency (91–99) *n* %)		4 (3.2%)		2 (2.2%)		2 (5.9%)
Moderate dependency (61–90) *n* (%)		16 (12.7%)		10 (10.9%)		6 (17.6%)
Severe dependency (21–60) *n* (%)		12 (9.5%)		7 (7.6%)		5 (14.7%)
Total dependency (0–20) *n* (%)		69 (54.8%)		50 (54.3%)		29 (55.9%)
Barthel index 90 days	123		89		34	
Independence (100) *n* (%)		43 (35%)		32 (36%)		11 (32.4%)
Slight dependency (91–99) *n* %)		3 (2.4%)		2 (2.2%)		1 (2.9%)
Moderate dependency (61–90) *n* (%)		11 (8.9%)		8 (9.0%)		3 (8.8%)
Severe dependency (21–60) *n* (%)		12 (9.8%)		8 (9.0%)		4 (11.8%)
Total dependency (0–20) *n* (%)		54 (43.9%)		39 (43.8%)		15 (44.1%)
Barthel index 180 days	98		68		30	
Independence (100) *n* (%)		34 (34.7%)		24 (35.3%)		10 (33.3%)
Slight dependency (91–99) *n* %)		2 (2%)		1 (1.5%)		1 (3.3%)
Moderate dependency (61–90) *n* (%)		6 (6.1%)		4 (5.9%)		2 (6.7%)
Severe dependency (21–60) *n* (%)		3 (3.1%)		0 (0%)		3 (10%)
Total dependency (0–20) *n* (%)		53 (54.1%)		39 (57.4%)		14 (46.7%)

Classification proposed by Shah et al. [21]

#### The dynamic SOFA score during the first 5 days was a predictor of high disability in non-COVID ARDS

**Fig. 4:**
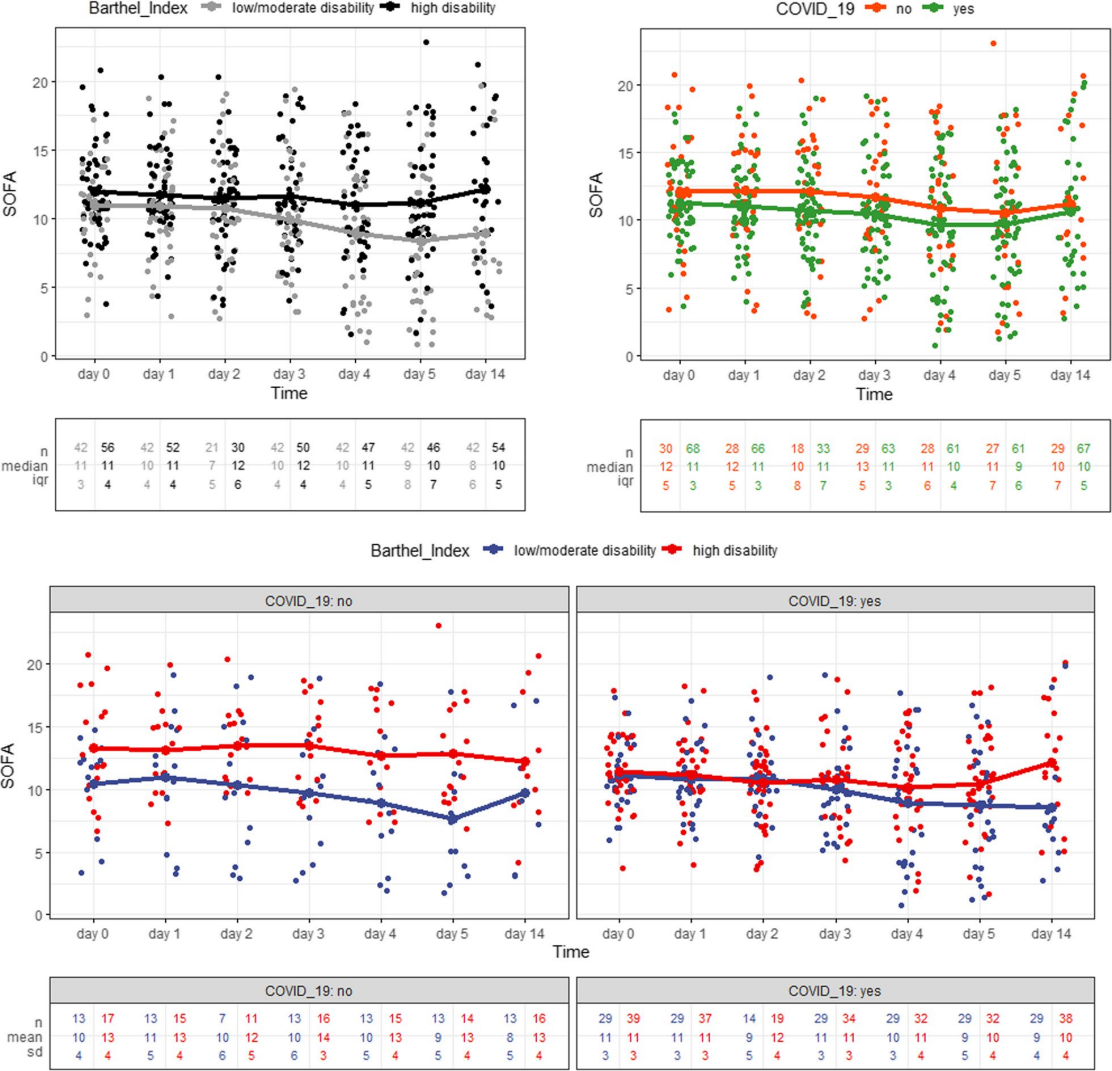
Line diagram of SOFA score stratified by Barthel index at 180 days and COVID-19 ARDS (*n* = 68) vs. Non-COVID-19 ARDS (*n* = 30) showing the dynamic of SOFA score development within the first 15 days after study enrollment

We also compared the 180-day SOFA score trajectories stratified by the BI and etiology of ARDS (COVID-19 ARDS and non-COVID ARDS) using a linear mixed model (Fig. 4 and Table 4). The mean SOFA score and mean changes in the SOFA score during the first 5 days after enrollment were greater in the high disability group at day 180 (main effect moderate/high disability at day 180 (β = 4.61, 95% CI 2.25–6.98, *P* =  < 0.001; interaction high disability at day 180: Time: β = 0.54, 95% CI 0.325–0.76, *P* < 0.001).

**Table 4:** Mixed effects model results showing the relationship between SOFA score, Barthel index at day 180, and COVID-19

	Beta (β)	95% CI	*P* value	
Intercept	8.65			AIC: 2875.2
Time	− 0.51	− 0.67; − 0.34	< 0.001	BIC 2892.8
Barthel high disability* (day 180)	4.61	2.25; 6.98	< 0.001	
COVID yes	0.51	− 1.52; 2.54	0.62	
Interaction Barthel high disability*: Time	0.54	0.32; 0.76	< 0.001	
Interaction COVID: Barthel high disability*	− 2.88	− 5.57; − 0.18	0.037	

To reduce collinearity, the variables COVID yes and Barthel index were labeled as follows: not: − 0.5 and yes: 0.5 and time was centered

*High disability: Barthel index 0–60

No differences were observed between the group COVID-19 ARDS vs. non-COVID ARDS on the mean SOFA score (*P* = 0.62) (Table 4). As the interaction between the Barthel index and the presence of COVID-19 was significant (*P* = 0.037), a subgroup analysis was performed by splitting the cohort based on COVID-19.

In patients without COVID-19 ARDS, low vs. high disability at day 180 was significantly positively associated with higher SOFA score over time (β = 3.52, 95% CI 0.69; 6.35, *P* = 0.017). In the COVID-19 group, moderate/high disability at day 180 was not significantly associated with a change in SOFA score (β = 1.01, 95% CI − 0.33; 2.36, *P* = 0.139) (Fig. 4 and Additional file 4: Table S3).

## Discussion

Long-term sequelae after ICU therapy in patients with ARDS have become an increasing subject of interest in recent years, especially since the COVID-19 pandemic. The question of whether COVID-19 ARDS is associated with a higher mortality rate and increased risk of long-term impairment compared to ARDS of other origins is widely discussed. Similar to previous findings [22, 23], the mortality of COVID-19 ARDS was not higher than that of non-COVID-19 ARDS in our study population. Unlike Sjoding et al., who performed a retrospective analysis, or Bain et al., whose non-COVID-19 cohort was retrospectively recruited, the ThIlo cohort was prospective.

Previous research has shown that patients with COVID-19 have a high prevalence of disability after ICU treatment [24], and the damage caused by COVID-19 ARDS is a problem that outlasts ICU treatment and brings a plethora of long-term health-related difficulties [25–27]. The question remains as to whether COVID-19 presents a higher risk of impairment. In a small cohort, Valent et al. showed that COVID-19 survivors had diminished SF-36 scores, which were lower than those in previously described patients with Middle East respiratory syndrome or influenza infection [28]. Our data reveal that COVID-19 ARDS was not associated with an increased risk of impairment compared to non-COVID ARDS after ICU discharge. Within the first 180 days, the percentage of patients remaining highly disabled, as displayed by the BI, did not differ, and recovery seems to take place at a comparable pace.

It is unquestionable that patients with ARDS are at risk of sustaining long-term impairment. Previous studies have described impaired quality of life and pulmonary function in patients with acute lung injury [5, 12, 24–26, 29–34] after ICU discharge.

The identification of risk factors for impairment after ARDS survival might provide the opportunity to intervene specifically to improve patient outcomes. Similar to previous studies, this study shows that age is a significant but non-amendable factor for disability after discharge [12, 30, 31, 34]. In addition, other factors such as obesity and malnutrition [27, 35], time interval of mechanical ventilation [35], renal replacement therapy, and length of stay have been discussed as significant factors affecting the quality of life after hospital discharge.

In addition to age, our findings revealed that health status on day 5 in ICU (reflected by the SOFA score) significantly impacted disability after discharge. Our results are consistent with Herridge et al. [30], who correlated the APACHE II score and any illness acquired during ICU stay with reduced walking distance at 6 and 12 months after discharge.

The SOFA score was chosen because it is an estimated marker for mortality in ICU patients [36, 37]. In our study, patients with high disability had significantly higher SOFA score components not only for respiration, but also for the nervous system and kidney failure. The SOFA components for liver failure and coagulation were also different, although they did not reach statistical significance (data not shown). Similar to Herridge et. al., we report faster resolution of organ failure results, unsurprisingly, in a better outcome [31]. The BI was chosen as it is a marker emphasizing functional and social activities in daily living and a well-described tool for evaluating the quality of life after ICU treatment [13]. A BI < 80, for instance, has been described as a good predictor of mortality in patients with CAP [38]. In our subgroup analysis, the SOFA score dynamic up to day 5 significantly influenced disability in patients without COVID-ARDS. Therefore, the first five days seem to be a key timeframe for long-term recovery after ARDS that is not caused by COVID-19.

For COVID-19 ARDS, the SOFA score dynamic up to day 5 was not significant.

Independent of the dynamic, however, the SOFA score on day 5 significantly influenced disability after ARDS in both groups. This shows the importance of the state of the patient on day 5.

However, the question arises if the outcome depends on the well-timed initiation of therapy [39] rather than on the rate of infection or organ replacement therapy, resulting in a longer ICU length of stay and/or longer and higher risk of disability. This might explain why patients with ARDS with low quality of life show a higher incidence of renal replacement therapy, which our data also reflect, and a longer stay on ICU in several studies. It remains to evaluate if striving to improve the patient’s status early-within the first five days-with more aggressive methods would result in a more beneficious outcome, or if the day 5 SOFA score can be used as a non-amendable outcome predictor only.

Our data are limited, as we did not evaluate patients’ health status prior to their ICU admission. Data on frailty, specifically, prior to ICU admission were not surveyed; however, it would have been of interest since frailty per se is a predictor for ICU  length of stay, length of mechanical ventilation, and mortality [40–42].

Our study was not primarily designed to define the quality of life or compare COVID-19 ARDS with ARDS of other origins, but is a secondary analysis of the interventional ThIlo trial.

The trial started in the spring of 2019, before the beginning of the COVID-19 pandemic, and lasted until spring of 2021. During the course of the pandemic, specific treatment of COVID-19 patients changed, and in the beginning varied from “wave to wave”. Therefore, systematic comparison of COVID-19 ARDS with non-COVID-ARDS is not as simple. All in all, there certainly were variables-also within the therapy-which differed over time, and were not systematically evaluated and compared. As data were collected up until spring of 2021, none of our COVID-19 patients were vaccinated. Therefore, our results might not reflect the current morbidity and mortality that COVID-19 ARDS brings with it, as vaccination and more specific COVID-19 treatment is available now.

In summary, the mortality of COVID-19 ARDS in our study population was not higher than that of ARDS of other origins and was not associated with an increased risk of disability. In both groups, early recovery from multiorgan dysfunction reduced disability after ICU discharge. The development of SOFA score within the first 5 days or SOFA score on day 5 significantly impacted patient outcome. Further research on the timing of intensive care treatment could help reduce mortality and health care costs in patients with ARDS.

## Supplementary Information

**Additional file 1**: **Fig. S1**. Receiver operating characteristic-ROC–for a high disability (Barthel Index between 0–60) after 180 days and SOFA score at day 5. (*n* = 98).

**Additional file 2. Supplemental Table 1:** Risk factors for high disability* at day 180 (multivariable analysis by binary logistic regression after multiple imputation n=144).

**Additional file 3. Supplemental Table 2:** Comparison of Barthel index at follow up in COVID-19 vs. Non-COVID-19 groups (ANOVA type test, after multiple imputation n = 144).

**Additional file 4. Supplemental Table 3:** Mixed effects model results showing the relationship between SOFA score and Barthel index at day 180.

## Data Availability

After publication, the data will be made available upon reasonable request from the corresponding author.
