# Supplementary figures and images for: COVID-19 does not influence functional status after ARDS therapy
Source: Crit Care. 2023 Feb 5;27:48. doi: 10.1186/s13054-023-04330-y (PMC9899507; doi:10.1186/s13054-023-04330-y)

## Slide 1
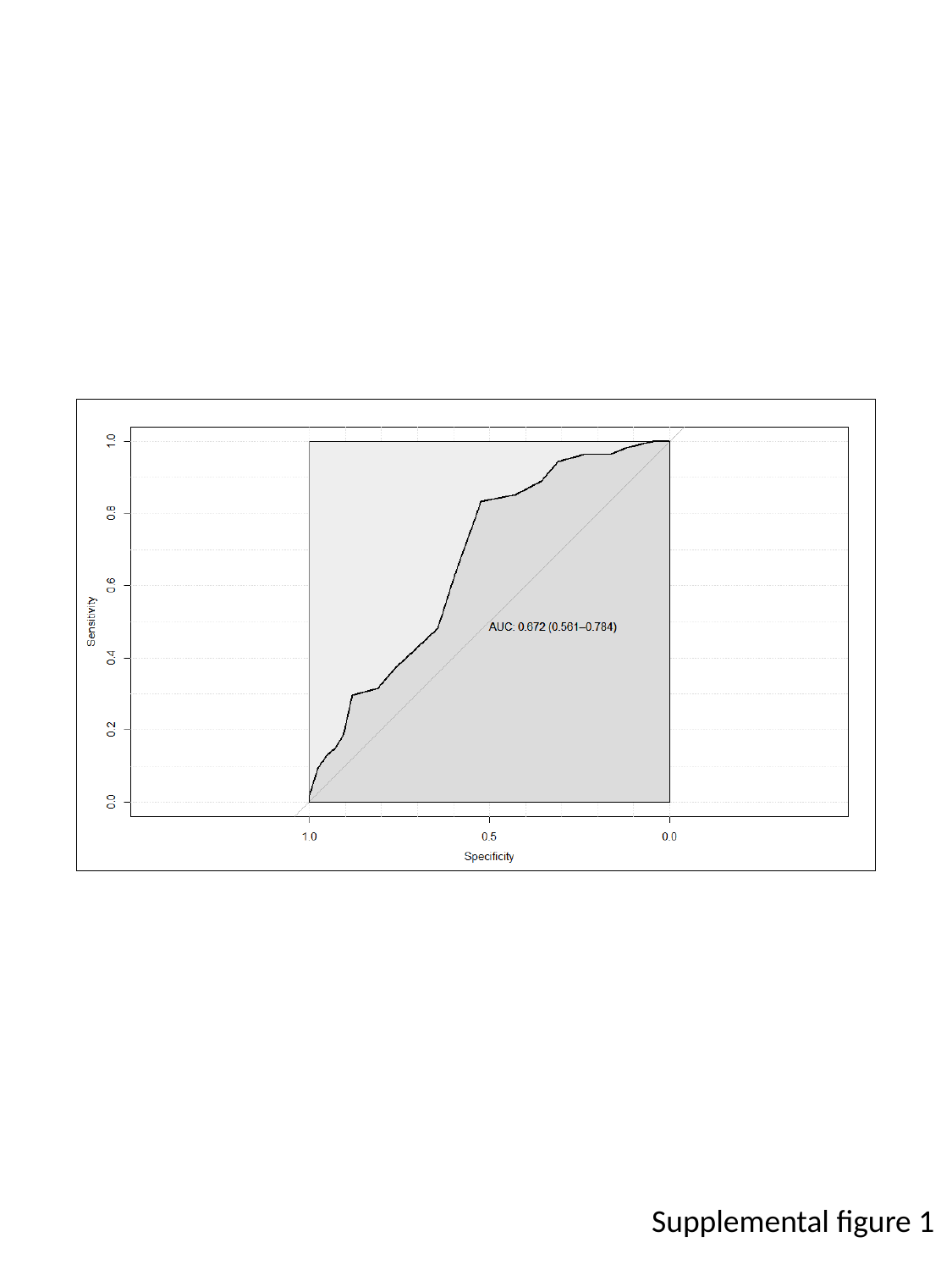

Supplemental figure 1

Supplement: Supplementary file 1 — Additional file 1: Fig. S1. Receiver operating characteristic-ROC–for a high disability (Barthel Index between 0–60) after 180 days and SOFA score at day 5. (n = 98). [file 13054_2023_4330_MOESM1_ESM.pptx]
