# Supplementary material for: COVID-19 does not influence functional status after ARDS therapy
Source: Crit Care. 2023 Feb 5;27:48. doi: 10.1186/s13054-023-04330-y (PMC9899507; doi:10.1186/s13054-023-04330-y)
Supplement: Supplementary file 2 — Additional file 2. Supplemental Table 1: Risk factors for high disability* at day 180 (multivariable analysis by binary logistic regression after multiple imputation n=144). [file 13054_2023_4330_MOESM2_ESM.docx]

**Supplemental Table 1:** Risk factors for high disability* at day 180 (multivariable analysis by binary logistic regression after multiple imputation n=144)

|  | OR | 95% CI | P value |
| --- | --- | --- | --- |
| *Variables at baseline assessment* | | | |
| Age in years | 1.05 | 1.01 – 1.09 | 0.015 |
| SOFA 5 days ICU | 1.12 | 1.01 – 1.25 | 0.043 |
| COVID – 19 yes | 0.66 | 0.21 – 2.09 | 0.44 |
| ECMO yes | 3.68 | 1.09 – 12.5 | 0.040 |

*high disability: Barthel index 0 - 60
