# Supplementary material for: COVID-19 does not influence functional status after ARDS therapy
Source: Crit Care. 2023 Feb 5;27:48. doi: 10.1186/s13054-023-04330-y (PMC9899507; doi:10.1186/s13054-023-04330-y)
Supplement: Supplementary file 3 — Additional file 3. Supplemental Table 2: Comparison of Barthel index at follow up in COVID-19 vs. Non-COVID-19 groups (ANOVA type test, after multiple imputation n = 144). [file 13054_2023_4330_MOESM3_ESM.docx]

**Supplemental Table 2: comparison of Barthel index at follow up in COVID-19 vs.** Non-COVID-19 groups (**ANOVA-type test**, after multiple imputation n = 144)

|  | Statistic (ANOVA-type statistic) | df | p-value |
| --- | --- | --- | --- |
| COVID-19 ARDS | 0.08 | 1 | 0.82 |
| Time | 64.9 | 2.43 | <0.001 |
| COVID-19 : Time | 0.82 | 2.43 | 0.51 |
