# Supplementary material for: COVID-19 does not influence functional status after ARDS therapy
Source: Crit Care. 2023 Feb 5;27:48. doi: 10.1186/s13054-023-04330-y (PMC9899507; doi:10.1186/s13054-023-04330-y)
Supplement: Supplementary file 4 — Additional file 4. Supplemental Table 3: Mixed effects model results showing the relationship between SOFA score and Barthel index at day 180. [file 13054_2023_4330_MOESM4_ESM.docx]

**Supplemental Table 3:** Mixed effects model results showing the relationship between SOFA score and Barthel index at day 180

|  | Beta (β) | 95% CI | P value |
| --- | --- | --- | --- |
| Cohort non-COVID (n = 44) | | | |
| Intercept | 10.05 |  |  |
| Time | -0.15 | -0.23 ; -0.06 | <0.001 |
| Barthel high disability (day 180) | 3.52 | 0.69 ; 6.35 | 0.017 |
| Cohort COVID-19 (n = 100) | | | |
| Intercept | 10.24 |  |  |
| Time | -0.11 | -0.17 ; -0.04 | 0.002 |
| Barthel high disability (day 180) | 1.01 | -0.33 ; 2.36 | 0.139 |
